# IL-15-dependent immune crosstalk between natural killer cells and dendritic cells in HIV-1 elite controllers

**DOI:** 10.1016/j.celrep.2023.113530

**Published:** 2023-12-03

**Authors:** Ciputra Adijaya Hartana, Melanie Lancien, Ce Gao, Yelizaveta Rassadkina, Mathias Lichterfeld, Xu G. Yu

**Affiliations:** 1 Ragon Institute of MGH, MIT, and Harvard, Cambridge, MA 02139, USA; 2 Infectious Disease Division, Brigham and Women’s Hospital, Boston, MA 02115, USA; 3 Broad Institute of MIT and Harvard, Cambridge, MA 02142, USA

**Keywords:** NK cells, HIV infection, elite controllers, trained innate immunity, IL-15, chromatin modifications, cytotoxicity, metabolism

## Abstract

As the principal effector cell population of the innate immune system, natural killer (NK) cells may make critical contributions to natural, immune-mediated control of HIV-1 replication. Using genome-wide assessments of activating and inhibitory chromatin features, we demonstrate here that cytotoxic NK (cNK) cells from elite controllers (ECs) display elevated activating histone modifications at the interleukin 2 (IL-2)/IL-15 receptor β chain and the *BCL2* gene loci. These histone changes translate into increased responsiveness of cNK cells to paracrine IL-15 secretion, which coincides with higher levels of IL-15 transcription by myeloid dendritic cells in ECs. The distinct immune crosstalk between these innate immune cell populations results in improved IL-15-dependent cNK cell survival and cytotoxicity, paired with a metabolic profile biased toward IL-15-mediated glycolytic activities. Together, these results suggest that cNK cells from ECs display a programmed IL-15 response signature and support the emerging role of innate immune pathways in natural, drug-free control of HIV-1.

## Highlights

• Enhanced activating histone marks on *IL2R*β and *BCL2* genes in cNK cells from ECs
• Elevated levels of IL-15 transcription in myeloid DCs from ECs
• Improved survival and increased cytotoxicity in cNK cells after IL-15 stimulation
• Increased glycolytic activities in cNK cells from ECs after IL-15 stimulation

## Graphical abstract

**Figure:**
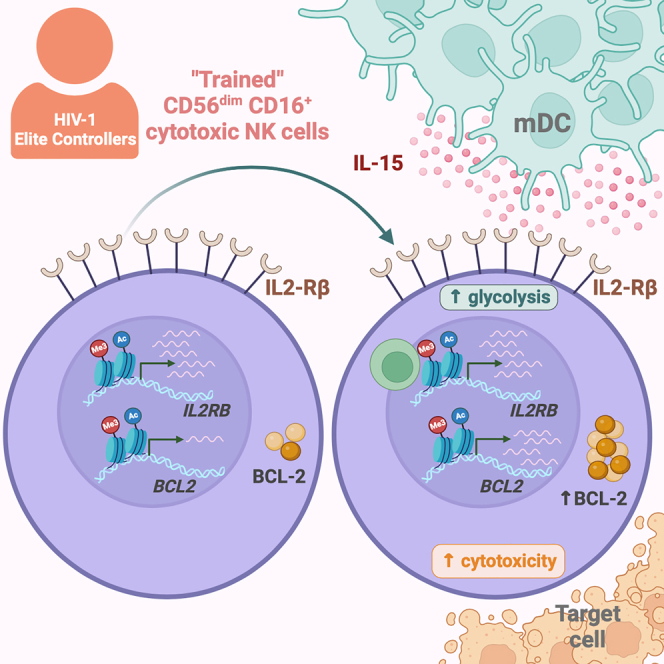

## Teaser

Hartana et al. observed elevated IL-15 responsiveness of cNK cells from HIV-1 elite controllers, paired with increased levels of IL-15 transcription in mDCs. These results suggest that immune crosstalk between mDCs and cNK cells results in distinct IL-15 response signatures and may contribute to drug-free control of HIV-1 replication.

## Introduction

A cure for HIV-1 infection, previously considered elusive, has been observed in four people living with HIV (PLWH) with leukemia undergoing transplantation with CCR5Δ32-expressing hematopoietic stem cells.^1^^,^^2^^,^^3^^,^^4^ Provocatively, the absence of intact HIV-1 proviruses in billions of peripheral blood mononuclear cells (PBMCs) has also been described in two PLWH without hematopoietic stem cell transplantation, suggesting that a “virological cure” of HIV-1 disease can emerge through natural, immune-mediated mechanisms.^5^^,^^6^ Drug-free, “elite” control of plasma HIV-1 viremia to undetectable levels, frequently considered a “functional” cure of HIV-1-infection, occurs in approximately 0.5%–1% of PLWH; however, small reservoirs of infected cells are readily detectable in these individuals.^7^ Although host immune factors are widely considered as the underlying etiology of elite control,^8^^,^^9^ insight into the specific underlying immune mechanisms remains limited. Recent studies suggest a bias of proviral HIV-1 integration sites toward heterochromatin chromosomal locations in elite controllers (ECs), best explained by immune selection pressure, promoting the elimination of proviruses in accessible chromatin.^5^ In contrast, proviruses integrated in non-accessible genomic regions and displaying a state of deep latency, presumably with a more limited ability to drive viral rebound, seem to persist long term in EC.^5^

HIV-1-specific CD8^+^ T cells are considered the most likely drivers of such immunological selection processes^10^^,^^11^^,^^12^^,^^13^^,^^14^ based on strong immunogenetic associations between polymorphisms within the HLA class I binding pocket and natural HIV-1 control.^10^ However, a role for innate immune mechanisms, in particular natural killer (NK) cells, in HIV-1 immune control has also been proposed.^15^ Like CD8^+^ T cells,^16^^,^^17^^,^^18^ NK cells undergo rapid and dynamic changes in the early phase of HIV-1 infection, and they can mount direct cytotoxic effects against HIV-1-infected cells that do not depend on HLA class I restriction and are less prone to viral mutational escape.^19^^,^^20^^,^^21^ Furthermore, early data suggested that the frequency of cytotoxic NK cells is almost two times higher in HIV-1 ECs compared to non-controller PLWH^22^ and that NK cells from ECs may have distinct phenotypic profiles.^23^ Moreover, NK cells can effectively suppress viral replication in *in vitro* viral inhibition assays^24^^,^^25^^,^^26^^,^^27^^,^^28^^,^^29^ independently of CD8^+^ T cells.^30^^,^^31^ Several subsets of NK cells are distinguished based on phenotypic and functional considerations. CD56^dim^ CD16^+^ cytotoxic NK (cNK) cells, which are investigated in this study, represent the most dominant subset of NK cells and have direct cytotoxic capacities.^19^ In contrast, CD56^bright^ CD16^−^ NK cells are known for their ability to produce inflammatory cytokines, providing paracrine activation signals to other immune cells.^19^ While some studies suggested the evolution of memory NK cell responses, predominantly in animal models,^32^^,^^33^^,^^34^^,^^35^ definitive evidence for NK memory cells in humans is currently lacking. Yet, several studies demonstrated that innate immune cells can be “trained.”^36^^,^^37^

The concept of “trained innate immunity” suggests that innate immune cells are poised by an initial viral infection to respond more potently upon secondary infections, in a pattern that is reminiscent of a memory-cell-like behavior.^38^ Trained innate immune cells can frequently be distinguished by distinct epigenetic profiles, which we define as histone modifications and other chromatin characteristics that impact gene transcription without changing the DNA sequence; frequently, epigenetic changes can prominently shape the cellular metabolism and functions of these cells.^17^^,^^36^^,^^39^^,^^40^ Our recent work suggests that myeloid dendritic cells (mDCs) from HIV-1 ECs have improved immunometabolic properties, likely as a result of epigenetic changes in gene-locus-specific histone modifications.^41^ These results raised the possibility that in ECs, trained innate immune responses can make critical contributions to immunological selection pressure against infected CD4^+^ T cells. In this study, we hypothesized that NK cell responses with features of “trained immunity” contribute to HIV-1 immune control in ECs. Based on a comprehensive characterization of epigenetic, transcriptomic, and metabolic profiles of CD56^dim^ CD16^+^ cNK cells from a well-characterized cohort of ECs, we demonstrated that NK cells from such individuals displayed distinct epigenetic and transcriptional features, which are likely to influence their survival, cytotoxic capacities, metabolic activities, and susceptibility to interleukin-15 (IL-15)-mediated immune crosstalk with DCs.

## Results

### Distinct epigenomic profiles of CD56^dim^ CD16^+^ cNK cells from ECs

**Figure 1:**
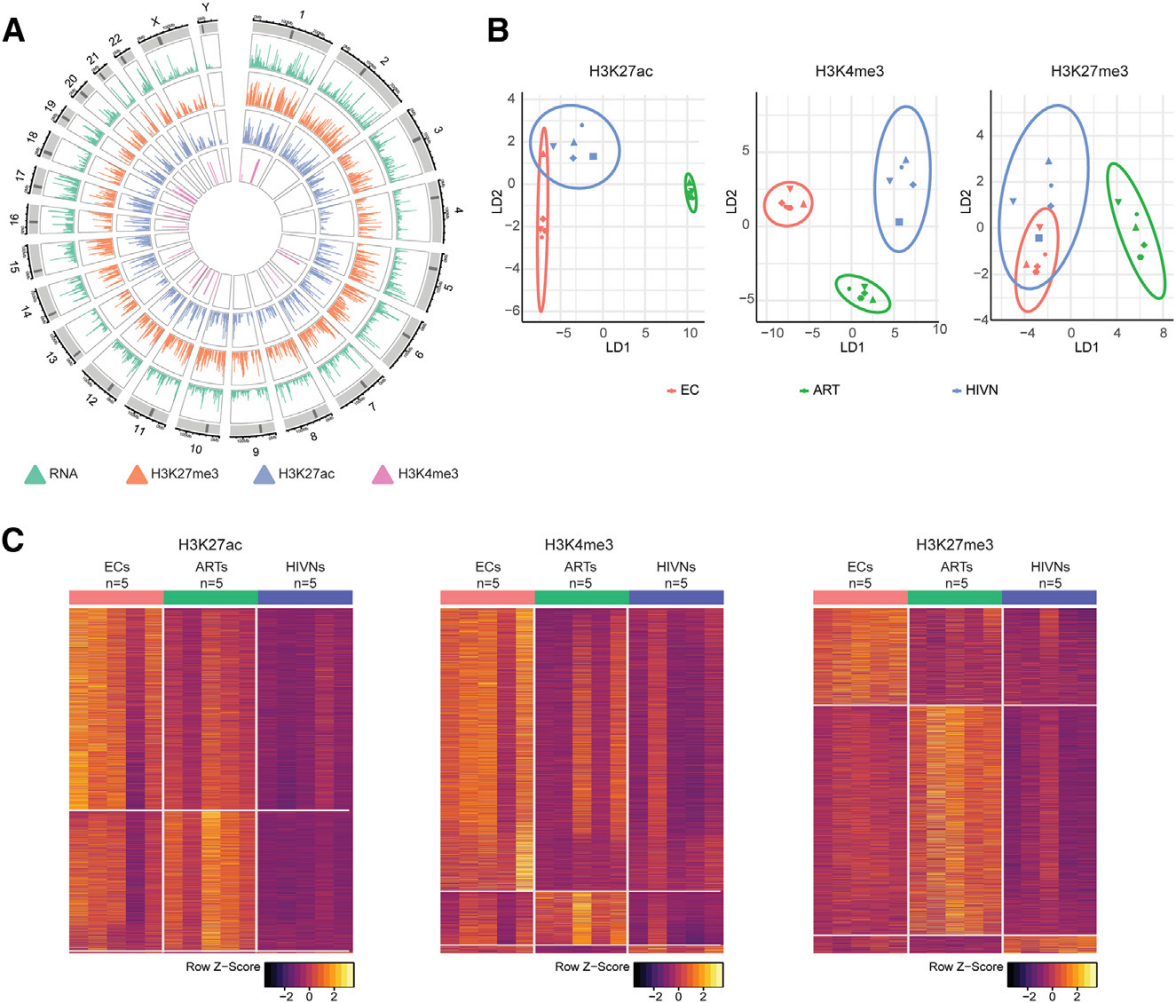
CD56^dim^ CD16^+^ cNK cells from ECs displayed distinct epigenomic profiles (A) Circos plots reflecting the enrichment profiles of histone H3K27me3, H3K27ac, and H3K4me3 marks from CUT&RUN-seq in relation to the transcriptomes from RNA-seq in all chromosomal locations in primary CD56^dim^ CD16^+^ cNK cells. (B) Linear discriminant analysis (LDA) of the epigenomic profiles of primary cNK cells for H3K27ac (left), H3K4me3 (middle), and H3K27me3 (right) marks in ECs (n = 5), ARTs (n = 5), and HIVNs (n = 5). Dot shape reflects study cohort. (C) Heatmap displaying genes with significantly enriched (p < 0.05) H3K27ac (left), H3K4me3 (middle), and H3K27me3 (right) marks in primary cNK cells from ECs (n = 5), ARTs (n = 5), and HIVNs (n = 5).

To evaluate NK cell profiles from ECs, we initially performed an epigenetic analysis of sorted primary NK cells from 5 ECs relative to PLWH treated with antiretroviral therapy (ART) (ARTs; n = 5) and people without HIV (HIVNs; n = 5) (Figure 1A). Epigenomic analysis was performed using the cleavage under target and release using nuclease sequencing (CUT&RUN-Seq) assay,^42^ allowing for a genome-wide identification of DNA segments binding to defined activating (H3K27ac and H3K4me3) or inhibitory (H3K27me3) histone marks. Given that epigenetic and transcriptional features may vary within individual NK cell subsets, we restricted our experiments to sorted CD56^dim^ CD16^+^ cNK cells, which possess strong cytotoxic activities and typically represent the most prevalent NK cell subset in adults.^19^ As an additional reference cell population, we sorted CD56^bright^ CD16^−^ NK cells, frequently characterized by secretions of inflammatory cytokines^19^ (Figure S1A).

We observed that the epigenomic profiles of activating H3K27ac and H3K4me3 marks in CD56^dim^ CD16^+^ cNK cells from ECs showed a discrete spatial positioning relative to the other two cohorts on linear discriminant analysis (LDA) plots (Figure 1B); in contrast, no such differences were observed for epigenetic profiles related to the inhibitory histone mark H3K27me3. Corresponding to this global analysis, we noted a signature of genes with significant enrichment or de-enrichment for H3K27ac and H3K4me3 at the respective genomic locations of cNK cells from ECs relative to the reference cell populations; however, genes with differential H3K27me3 marks were less obvious in ECs compared to people without HIV (Figure 1C; Table S1). Moreover, Ingenuity Pathway Analysis (IPA) suggested that genes with enhanced activating histone modifications in ECs were functionally involved in the regulation of immune activity, oncogenesis, and cell metabolism (Figures S2A–S2F). In contrast to CD56^dim^ CD16^+^ cNK cells, epigenetic differences between ECs and reference cohorts of PLWH on ART or people without HIV were less apparent within in the CD56^bright^ CD16^−^ NK cell subset (Figure S1B). For this reason, our subsequent analysis focused on a further exploration of the CD56^dim^ CD16^+^ cNK cell population.

### Transcriptional and epigenetic activation of the IL-2-Rβ chain in cNK cells from ECs

**Figure 2:**
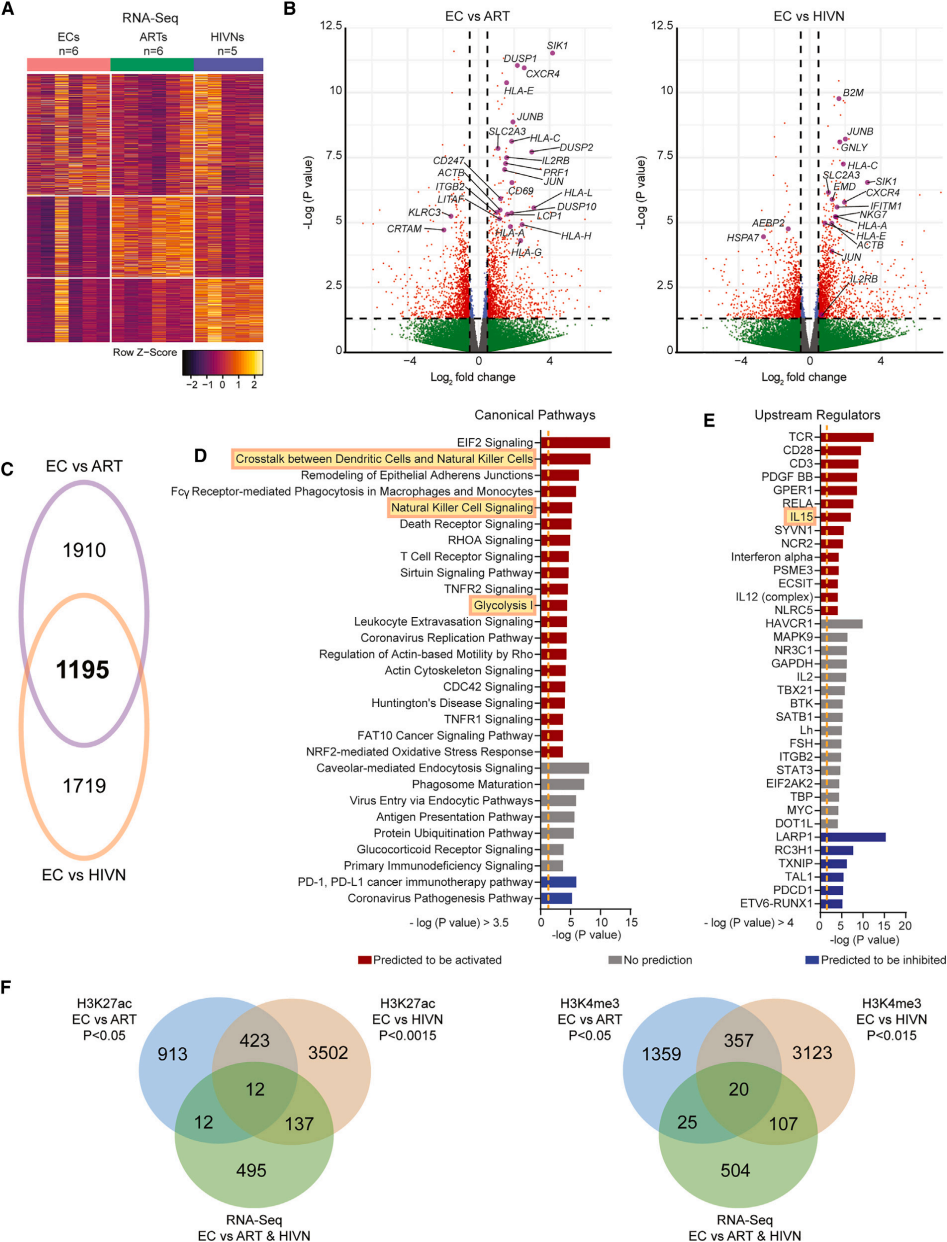
Transcriptional signatures of CD56^dim^ CD16^+^ cNK cells from ECs (A) Heatmap displaying differentially expressed genes (p < 0.05) in primary CD56^dim^ CD16^+^ cNK cells from ECs (n = 6), ARTs (n = 6), and HIVNs (n = 5) using RNA-seq analysis. (B) Volcano plot showing differentially expressed genes in primary cNK cells from ECs (n = 6) in comparison to ARTs (n = 6; left) and HIVNs (n = 5; right). Red dots represent data with –log (p value) >1.3 and log2 fold change >0.5 or < −0.5. Selected genes with relevant NK cell function involvement are highlighted. (C) Venn diagram showing the overlaps between differentially expressed genes (p < 0.05) between ECs vs. HIVNs and ECs vs. ARTs. (D and E) Significant canonical pathways (D) and upstream regulators (E) predicted by Ingenuity Pathway Analysis (IPA) of differentially expressed genes from (A). Predicted up- and down-regulated pathways are marked in red and blue, respectively. Pathways with no predicted changes are marked in gray. Orange dashed lines mark –log (p value) = 1.3. (F) Venn diagrams showing the overlaps between genes with increased histone H3K27ac (left) and H3K4me3 (right) enrichment in primary cNK cells between ECs vs. HIVNs and ECs vs. ARTs on the upregulated genes (p < 0.05).

We subsequently evaluated the associations between the epigenetic profiles and transcriptional signatures. For this purpose, we performed RNA-Seq on autologous sorted CD56^dim^ CD16^+^ cNK cells from our study participants. Overall, we noted a number of genes that were differentially expressed in ECs relative to PLWH on ART and people without HIV (Figures 2A and 2B). A computational IPA predicted that this differential gene expression signature in cNK cells from ECs (Figure 2C) translated into activation of pathways related to the crosstalk between DCs and NK cells, NK cell signaling, metabolic regulation, and IL-15-dependent immune activation (Figures 2D and 2E).

**Figure 3:**
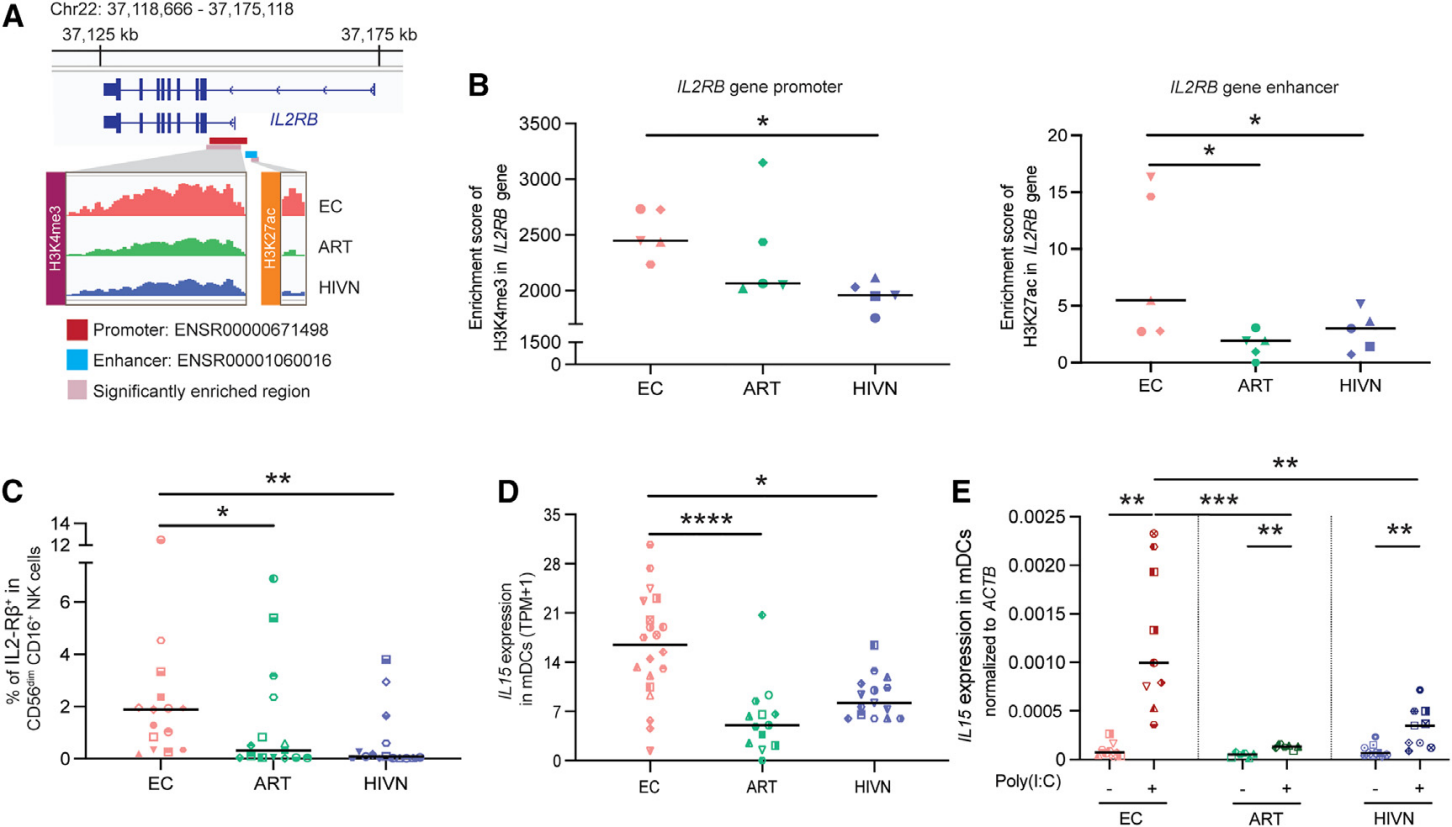
CD56^dim^ CD16^+^ cNK cells from ECs are supported by mDC-NK cell crosstalk via IL-15-IL-2-Rβ axis (A) CUT&RUN-seq reads for H3K27ac and H3K4me3 marks at the *IL2RB* gene locus in primary CD56^dim^ CD16^+^ cNK cells from a representative EC, ART, and HIVN. Purple areas mark regions with significantly increased histone H3K27ac or H3K4me3 reads. Predicted promoter and enhancer regions from Ensembl database are highlighted in red and blue, respectively. (B) The enrichment scores for H3K4me3 and H3K27ac marks from the CUT&RUN-seq reads in the promoter (left) and enhancer (right) regions, respectively, of the *IL2RB* gene were compared between ECs, ARTs, and HIVNs (n = 5 in each cohort). Kruskal-Wallis test was used for statistical analysis. (C) The frequencies of IL-2-Rβ^+^ cNK cells were compared among ECs (n = 15), ARTs (n = 15), and HIVNs (n = 15). Kruskal-Wallis test was used for statistical analysis. (D) The mRNA expression (TPM+1) of *IL15* in mDCs was compared among ECs (n = 20), ARTs (n = 13), and HIVNs (n = 15). Kruskal-Wallis test was used as the statistical test. (E) *IL15* transcript expression normalized to *ACTB* in mDCs from ECs (n = 9), ARTs (n = 6), and HIVNs (n = 9), after 2 μg/mL poly(I:C) stimulation for 24 h or without stimulation, was measured by RT-PCR. Wilcoxon matched-pairs signed-rank test was used to compare *IL15* expression between stimulated and unstimulated cells, whereas Kruskal Wallis test was used to compare *IL15* expression among cohorts. Horizontal lines in (B)–(E) represent medians. ^∗^p < 0.05, ^∗∗^p < 0.01, ^∗∗∗^p < 0.001, ^∗∗∗∗^p < 0.0001.

To further investigate these transcriptional changes in the context of the epigenetic data, we focused on the gene encoding for the β chain (CD122) of the IL-2 and IL-15 receptor complex. CD122 (IL-2-Rβ) can form a heterodimer receptor complex for both IL-2 and IL-15 that is selectively expressed on cNK cells and CD8^+^ T cells.^43^ We observed that primary cNK cells from ECs had a higher enrichment of H3K27ac marks on the *IL2RB* gene region compared to PLWH on ART and people without HIV; higher H3K4me3 mark enrichments were also observed in ECs but reached levels of statistical significance only in comparison to people without HIV (Figures 2F, 3A, and S3A; Tables S2 and S3). Inhibitory H3K27me3 histone modifications showed no differences at the *IL2RB* locus between the three study cohorts. Notably, the enrichment of activating histone marks was seen in the promoter and enhancer regions of the *IL2RB* gene, which are essential for gene transcription (Figures 3A and 3B). Enhanced activating epigenetic marks on the *IL2RB* gene in ECs were associated with elevated transcriptional activity, specifically in comparison to people without HIV (Figures 3A and 3B). Consequently, an upregulation of the IL-2-Rβ surface protein expression was seen in primary, steady-state cNK cells from ECs compared to PLWH on ART (p = 0.0484) and people without HIV (p = 0.0018) (Figures 3C and S3B). Together, these findings suggest an altered epigenetic and transcriptional program in cNK cells from ECs that involves increased signaling via the IL-2-Rβ chain.

### Increased transcriptional propensity for paracrine IL-15 secretion in ECs

IL-2 and IL-15 both act as physiological ligands for the receptor complex formed by the IL-2 receptor β chain on NK cells^43^; however, IL-15 provides more potent signals for NK cell development, expansion, and survival compared to IL-2.^44^^,^^45^^,^^46^^,^^47^ Additionally, IL-15 seems to have a higher affinity to its receptor complex compared to IL-2.^45^ Using RNA sequencing (RNA-seq) data from defined sorted immune cell subsets from ECs,^48^ we noted that the expression of the *IL15* mRNA was significantly higher in mDCs from ECs compared to PLWH on ART (p < 0.0001) and people without HIV (p = 0.0186) (Figure 3D); a similar, although less pronounced, finding was made for CD4^+^ T cells but not for B cells or monocytes (Figure S3D). Following TLR3 stimulation using poly(I:C) to activate mDCs, the expression of *IL15* mRNA in mDCs from ECs increased profoundly and again markedly exceeded corresponding levels in mDCs from PLWH on ART (p = 0.0001) or people without HIV-1 (p = 0.0069) (Figure 3E). In contrast, *IL2* mRNA expression was not different in any of the immune cell subsets from any of our study cohorts (Figures 3E and S3C). Thus, the elevated epigenetic and transcriptional activation of the IL-2-Rβ chain in cNK cells from ECs seems to converge with an increased propensity of autologous mDCs (and, to a lesser extent, of CD4^+^ T cells) for paracrine IL-15 secretion. Together, these results suggest that improved IL-15 receptor/ligand interactions may underlie distinct cNK cell functionalities in ECs.

### cNK cells from ECs are epigenetically poised for improved BCL-2 upregulation and increased cytotoxicity after IL-15 stimulation

**Figure 4:**
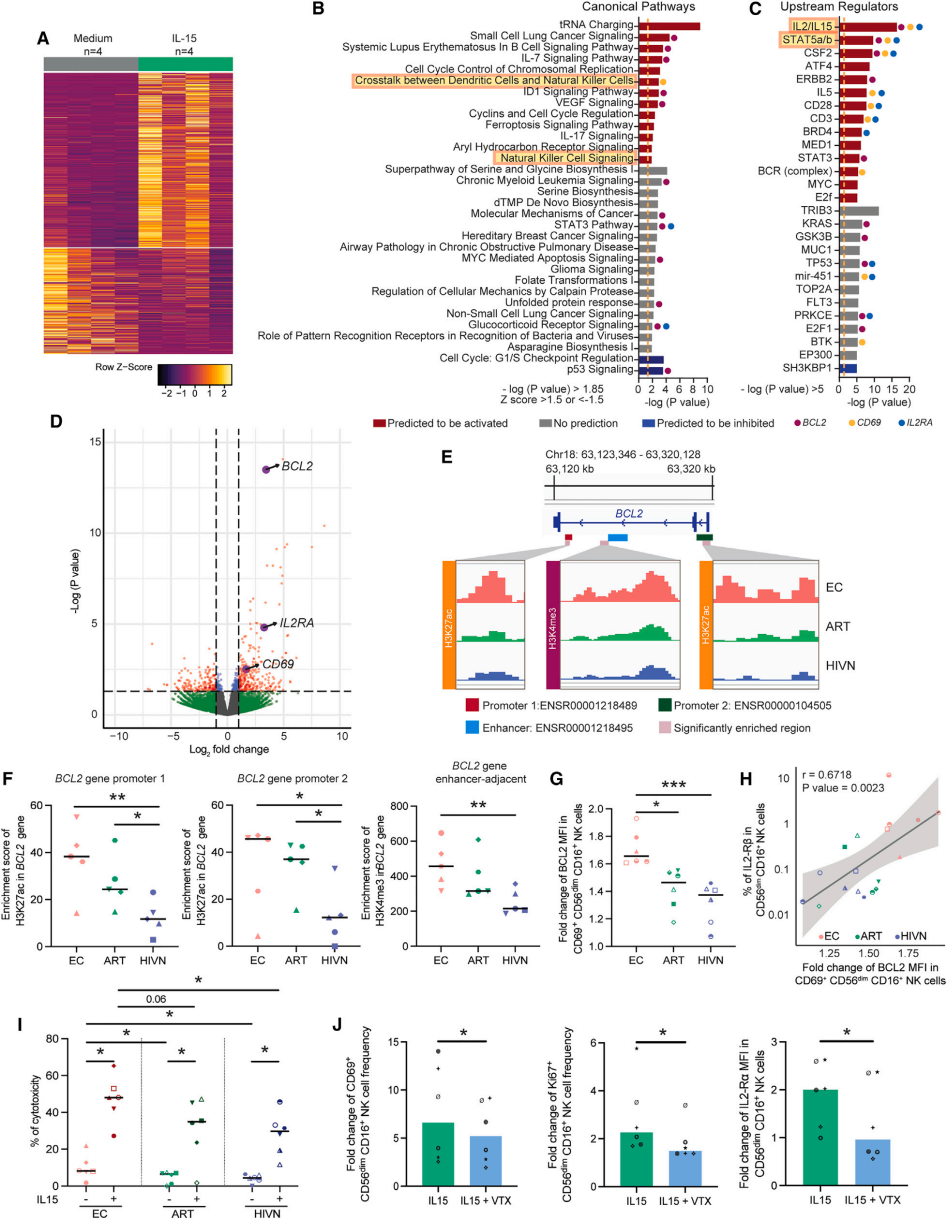
Epigenetic profiles and functional changes in CD56^dim^ CD16^+^ cNK cells after IL-15 stimulation (A) Heatmap displaying differentially expressed genes (p < 0.05) in NK cells, with or without 10 ng/mL IL-15 stimulation for 24 h using RNA-seq analysis. (B and C) Significant canonical pathways (B) and upstream regulators (C) predicted by IPA of differentially expressed genes from (A). Predicted up- and down-regulated pathways are marked in red and blue, respectively. Pathways with no predicted change are marked in gray. Orange dashed lines mark –log (p value) = 1.3. Canonical pathways and upstream regulators containing *BCL2*, *CD69*, and *IL2RA* are marked with purple, yellow, and blue dots, respectively. (D) Volcano plot showing differentially expressed genes in IL-15-stimulated vs. unstimulated NK cells. Red dots represent data with –log (p value) >1.3 and log2 fold change >1 or < −1. (E) CUT&RUN-seq reads for H3K27ac and H3K4me3 marks at the *BCL2* gene locus in primary CD56^dim^ CD16^+^ cNK cells from a representative EC, ART, and HIVN. Purple areas mark regions with significantly increased histone H3K27ac or H3K4me3 reads. Predicted promoter 1, promoter 2, and enhancer regions from Ensembl database are highlighted in red, green, and blue, respectively. (F) The enrichment scores for H3K27ac and H3K4me3 marks from the CUT&RUN-seq reads in the promoter- (left and middle) and enhancer-adjacent (right) regions, respectively, of the *BCL2* gene were compared between ECs, ARTs, and HIVNs (n = 5 in each cohort). Kruskal-Wallis test was used for statistical analysis. (G) The mean fluorescent intensities (MFIs) of BCL2 in CD69^+^ cNK cells were compared among ECs (n = 6), ARTs (n = 6), and HIVNs (n = 6). The MFIs are shown as fold changes after 10 ng/mL IL-15 stimulation relative to unstimulated controls. Kruskal-Wallis test was used for statistical analysis. (H) Correlation analysis was shown between the fold changes of BCL2 MFI in CD69^+^ cNK cells with the frequency of IL-2-Rβ^+^ cNK cells using Spearman test. (I) The cytotoxic activity of cNK cells against the K562 cell line was compared among ECs (n = 6), ART (n = 6), and HIVNs (n = 6), with and without IL-15 stimulation (10 ng/mL). Mann-Whitney and Wilcoxon matched-pairs signed-rank tests were used for statistical analysis. (J) The frequencies of CD69^+^ cNK cells and Ki-67^+^ cNK cells, as well as the MFI of IL-2Rα in cNK cells, were compared in cells stimulated with 10 ng/mL IL-15, with (n = 6) or without 100 nM venetoclax (n = 6) for 24 h. The data are shown as fold changes relative to unstimulated controls. Wilcoxon matched-pairs signed-rank test was used as the statistical test. Horizontal lines in (F)–(I) and bars in (J) represent medians. ^∗^p < 0.05, ^∗∗^p < 0.01, ^∗∗∗^p < 0.001.

To investigate the responses of NK cells to IL-15 stimulation, we performed transcriptomic profiling of NK cells from 4 people without HIV using RNA-seq. Consistent with the results of cNK cells analyzed directly *ex vivo* (Figures 2D and 2E), we noticed that the exposure to exogenous IL-15 resulted in differential expression of genes (Figure 4A) involved in pathways related to the crosstalk between DCs and NK cells, NK cell signaling, and IL-15-dependent immune activation (Figures 4B and 4C). Notably, the gene encoding for the antiapoptosis molecule BCL-2 was among the genes most strongly upregulated in IL-15-stimulated NK cells (Figures 4D and S4A). Moreover, predicted canonical pathways and upstream regulators responding to IL-15 stimulation frequently included the *BCL2* gene (Figures 4B and 4C). Other genes with notable upregulation following IL-15 stimulation encoded for the IL-2 receptor α chain and for the NK cell activation marker CD69 (Figures 4B–4D and S4A).

Notably, we observed an enrichment of the activating H3K27ac and H3K4me3 histone marks at the *BCL2* gene in cNK cells from ECs analyzed directly *ex vivo* compared to PLWH on ART and people without HIV (Figures 4E and S4B). These activating histone marks populated the promoter- and enhancer-adjacent regions of *BCL2* gene (Figures 4E and 4F). In contrast, expression of *BCL2* in primary cNK cells from ECs was not elevated at baseline on the RNA or protein level (Figure S4C), suggesting that the activating histone marks at the *BCL2* gene locus in *ex*-*vivo*-isolated cNK cells are not directly translating into transcriptional activation but predispose cNK cells to increased upregulation of BCL-2 in response to IL-15 stimulation. To further explore this, we measured the intracellular expression of the BCL-2 protein following IL-15 stimulation in our study cohorts using flow cytometry. After IL-15 stimulation, intracellular BCL-2 expression was significantly higher in activated CD69^+^ cNK cells from ECs in comparison to the reference cohorts (ECs vs. ARTs p = 0.011; ECs vs. HIVNs p = 0.001) (Figure 4G). In addition, the expression changes of BCL-2 in CD69^+^ cNK cells after IL-15 stimulation were positively associated with the surface expression of the IL-2-Rβ chain at baseline (Figure 4H), suggesting that the elevated expression of the IL-15 receptor may contribute to a higher responsiveness to IL-15 stimulation, which then translates into elevated BCL-2 expression. Together, these findings suggest that elevated expression of the IL-2-Rβ chain synergizes with increased paracrine IL-15 secretion to enhance BCL-2 expression in epigenetically poised cNK cells. Such a distinct interplay between IL-15, the IL-2-Rβ chain, and the BCL-2 at the transcriptional and epigenetic levels is likely to translate into an improved survival of cNK cells from ECs.

Supporting this concept on a functional level, we observed that the proportion of cNK cells from ECs that remained viable over 24 h of *in vitro* culture with exogenous IL-15 was significantly higher compared to cNK cells from PLWH on ART and people without HIV (Figure S4D). We also noted that treatment with venetoclax, a BCL-2 inhibitor, resulted in a notable reduction in the proportion of CD69-, Ki-67-, and IL-2-Rα-expressing cNK cells, consistent with the important role of BCL-2 for maintaining the viability of cNK cells (Figure 4J). Interestingly, functional assays revealed that IL-15-stimulated cNK cells from ECs display significantly higher cytotoxic activities compared to people without HIV; there was also a trend for elevated cytotoxicity of IL-15-stimulated cNK cells from EC relative to PLWH on ART (Figure 4I). These results suggest that a higher responsiveness to IL-15 can translate into improved NK-cell-mediated killing of target cells. Together, these experiments support our hypothesis that the described epigenetic changes in cNK cells from ECs are functionally relevant for maintaining cell survival and cytotoxic activities.

### NK cells from ECs show increased glycolytic activities

**Figure 5:**
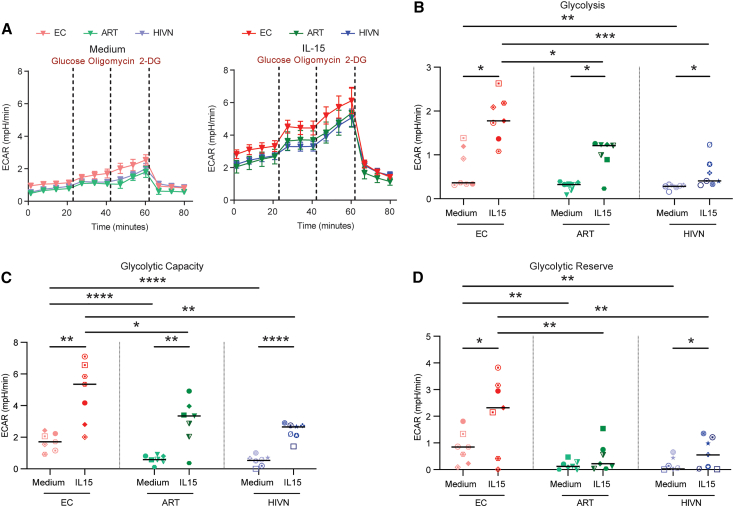
Increased IL-15-dependent glycolysis in NK cells from ECs (A) Extracellular acidification rate (ECAR) of NK cells from ECs (n = 7), ARTs (n = 7), and HIVNs (n = 7) with or without 10 ng/mL IL-15 stimulation for 24 h was measured at indicated time points in response to glucose, oligomycin, and 2-DG using a Seahorse XFe96 Analyzer. (B–D) Glycolysis (B), glycolytic capacity (C), and glycolytic reserve (D) of NK cells were compared in ECs, ARTs and HIVNs with or without 10 ng/mL IL-15 stimulation for 24 h. Paired t test was used to compare ECARs between stimulated and unstimulated cells, whereas one-way ANOVA test was used to compare ECARs among cohorts. Horizontal lines in (B)–(D) represent medians. ^∗^p < 0.05, ^∗∗^p < 0.01, ^∗∗∗^p < 0.001, ^∗∗∗∗^p < 0.0001.

Epigenetic changes in immune cells, specifically in the context of “trained immunity,” are frequently associated with altered metabolic programs.^38^^,^^49^ To further investigate functional changes in NK cells from ECs after IL-15 stimulation, we therefore evaluated their metabolic profiles, in particular their glycolytic activities. For this purpose, we stimulated NK cells from these cohorts with IL-15, followed by an evaluation of the extracellular acidification rate (ECAR), using the Seahorse technology.^50^ In response to IL-15 stimulation, a general increase of the ECAR was seen compared to baseline control (medium) (Figure 5A). Notably, we observed that glycolysis, glycolytic capacity, and glycolytic reserve were significantly higher in NK cells from ECs compared to PLWH on ART and people without HIV following IL-15 stimulation (Figures 5B–5D). These data suggest that NK cells from ECs display a distinct functional metabolic profile characterized by increased glycolytic activities following IL-15 stimulation.

## Discussion

Drug-free maintenance of undetectable plasma HIV-1 viral loads is best exemplified by ECs, and these individuals are frequently considered a model for a functional cure of HIV-1 infection. Innate immune activity may play an important role in promoting such a remarkable disease outcome, although their contribution relative to adaptive HIV-1-specific T cell-mediated immune responses remains an area of active investigation. Here, we demonstrated distinct epigenomic, transcriptomic, and metabolic profiles of CD56^dim^ CD16^+^ cNK cells from ECs. In particular, we found that primary cNK cells from ECs displayed higher levels of activating epigenetic chromatin features at the *IL2RB* gene locus, which was associated with a higher expression of the β chain of IL-15 receptor complex (IL-2-Rβ). Correspondingly, IL-15 transcription was strongly upregulated in mDCs from ECs, suggesting that the elevated expression of the IL-15 receptor on NK cells and enhanced paracrine IL-15 secretion in mDCs synergize in maintaining an enhanced IL-15 response signature in cNK cells from ECs. In line with these observations, we noted that IL-15 stimulation strongly enhanced BCL-2 protein expression and survival in cNK cells from ECs and augmented their cytotoxic properties and their functional glycolytic metabolic activities.

In previous studies, host characteristics of ECs were frequently evaluated using transcriptomic studies^51^^,^^52^^,^^53^^,^^54^ or genome-wide association studies (GWASs),^10^ while epigenetic investigations of immune cells, including NK cells, remain limited. Although epigenetic changes may in many cases not directly associate with gene expression changes at the transcriptional or proteomic level, they frequently can influence host behavior in response to immunological or microbiological challenges and contribute to a state of heightened innate immune activity frequently referred to as “trained immunity.”^38^ In our study, we observed at least two epigenetic alterations in cNK cells from ECs that likely influence their functional responsiveness and activity. We noted increased activating histone modifications at promoter and enhancer regions of the IL-2/IL-15 receptor β chain locus and at the *BCL2* gene locus. Both of these epigenetic changes seem functionally interconnected and may translate into increased functionality and resilience. Epigenetic and transcriptional activation of the IL-15 receptor can increase cell responsiveness to IL-15; IL-15 stimulation can then capitalize on epigenetically programmed BCL-2 activation to augment NK cell survival via BCL-2 upregulation. Together, these results support the hypothesis that epigenetic changes may predispose cNK cells to improved biological activity under conditions of stress and immune stimulation. The exact mechanisms that contribute to the evolution of trained immunity in cNK cells from ECs remains unknown at present but may involve prior pathogen exposure, specific immunogenetic alterations, or a fine-tuned cytokine milieu.^55^ Future efforts may allow for delineating epigenetic changes of cNK cells in much greater detail and may soon permit evaluation of the epigenetic and transcriptional changes of cNK cells at single-cell resolution.

Our data suggest qualitatively distinct cNK cell responses in ECs, primarily characterized by increased IL-15-dependent functionality that was paired with an increased IL-15 transcription in mDCs. We have previously shown that mDCs from ECs also present features of trained immunity,^41^ suggesting that innate immune activity in ECs is more broadly influenced by trained immune features. Of note, the higher constitutive levels of IL-15 in mDCs reported in this study could also be one functional consequence of the epigenetic remodeling of these cells, although this remains conjectural at the current time; in fact, our prior study failed to demonstrate significant upregulation of activating histone modifications at the IL-15 gene locus. Interestingly, IL-15 is currently being evaluated as an immune-modulatory agent in multiple clinical studies designed to increase drug-free control of HIV-1 infection. In humans, the pharmacological administration of the IL-15 super agonist N-803 resulted in an expansion of NK cell frequencies and activation, along with a modest reduction in inducible HIV-1 reservoir cells.^56^ Moreover, in simian immunodeficiency virus (SIV)-infected rhesus macaques, N-803 administration induced activation and trafficking of NK cells to the B cell follicles in lymph nodes,^57^ while inhibition of IL-15 using neutralizing IL-15 antibodies in SIV-infected rhesus macaques profoundly depleted NK cell numbers in peripheral blood and tissues.^58^ Finally, partial control of SIV after vaccination with a cytomegalovirus (CMV)-vectored immunogen was associated with a transcriptional signature dominated by IL-15 as an upstream regulator,^59^ a notion that corresponds well to the epigenetically programmed, IL-15-dependent signature of cNK cell regulation in ECs described here. One limitation of our current study was that IL-15 plasma levels were not directly assessed, given that the physiological half-life of IL-15 is extremely short,^60^ rendering it difficult to correctly measure IL-15 plasma concentrations. Moreover, we only analyzed the effects of IL-15 on NK cells in the peripheral blood, not in the lymph node samples, where IL-15 immune effects on NK cells may be most visible; therefore, future studies to investigate the role of IL-15 on NK cell trafficking to the lymph nodes in ECs will be informative.

An expanding set of data suggests that NK cells can contribute to immune defense against HIV-1 in controllers and post-treatment controllers,^15^ although their antiviral functions are less clearly understood compared to HIV-1-specific T cells. Some studies suggested that in ECs, the frequencies of CD56^dim^ cNK cells are two times higher, paired with lower expression of inhibitory markers TIGIT and LAG3.^22^ Other studies suggested that NK cells from ECs have higher expression levels of the activating receptor NKG2D and the cytotoxicity-associated marker CD57 and a lower expression of the inhibitory receptor NKG2A relative to NK cells from individuals without HIV.^26^ Generally, cytotoxic activities of NK cells are regulated by a specific set of activating and inhibitory receptors expressed on the NK cell surface and their corresponding ligands on target cells.^61^^,^^62^ Previous studies demonstrated that education of NK cells via KIRs or NKG2A increases their cytotoxic properties^63^^,^^64^; however, their true cytotoxic activity against HIV-1-infected cells is difficult to assess, given that the physiological expression of NK cell ligands on virally-infected, participant-derived CD4 T cells is not known. However, recent technical advances in single-cell proteogenomic profiling of HIV-1-infected cells may soon allow for jointly characterizing the cytotoxic activities of NK cells and the corresponding susceptibility of infected target cells to NK-cell-mediated killing.^65^ Such novel technologies will better permit determining whether the distinct epigenetic and transcriptional features of cNK cells from ECs translate into an improved functional effector cell profile.

In summary, the work presented here suggests that cNK cells from ECs display distinct epigenetic and transcriptional features that promote improved susceptibility to IL-15, which then translate into elevated IL-15-dependent survival and enhanced cytotoxic and metabolic activities. Such an altered functional state appears to resemble a condition of “trained innate immunity.” Together with prior studies demonstrating features of trained immunity in mDCs from ECs,^66^ our data suggest that altered epigenetic and transcriptional programs in innate immune cells may represent an important aspect of antiviral immune defense in ECs. Continued progress in understanding and unraveling the fascinating components of antiviral immune activity in these specific individuals may lead to actionable interventions designed to induce natural control of HIV-1 in larger numbers of individuals.

### Limitations of the study

We acknowledge that the number of study subjects reported in this article is relatively small; however, genome-wide epigenetic profiling can, at the current stage of technology development, not be conducted for larger numbers of study participants. Moreover, our data also failed to demonstrate direct mechanistic linkages between metabolic activities and cytotoxic activities of NK cells; such investigations will require dedicated future mechanistic evaluations.

## STAR★Methods

### Key resources table

**Table:** 

REAGENT or RESOURCE	SOURCE	IDENTIFIER
**Antibodies**		

Anti-CD3 PE-Cy5	BioLegend	AB_314046
Anti-CD3 APC-Cy7	BioLegend	B_314054
Anti-CD3 AF700	BioLegend	AB_493739
Anti-CD14 PE-Cy5	BioLegend	AB_2860767
Anti-CD14 APC-Cy7	BioLegend	AB_830693
Anti-CD14 AF700	BioLegend	AB_830687
Anti-CD19 PE-Cy5	BioLegend	AB_314240
Anti-CD19 APC-Cy7	BioLegend	AB_314248
Anti-CD19 AF700	BioLegend	AB_493751
Anti-CD56 BV570	BioLegend	AB_2563837
Anti-CD56 BV711	BioLegend	AB_2562417
Anti-CD56 BV650	BioLegend	AB_2563838
Anti-CD16 BUV395	BD Biosciences	Cat #: 563785
Anti-CD16 PE	BioLegend	AB_314208
Anti-CD122 (IL-2Rβ) BV786	BD Biosciences	Cat #: 743118
Anti-CD69 BUV805	BD Biosciences	Cat #: 748763
Anti-BCL2 PE	BioLegend	AB_2563282
Anti-Perforin PE-Cy7	BioLegend	AB_2571973
Anti-Granzyme B BV510	BD Biosciences	Cat #: 563388
Anti-Ki-67 BUV737	BD Biosciences	Cat #: 567130
Anti-CD25 (IL-2Rα) BUV563	BD Biosciences	Cat #: 612918
Anti-CD94 BB790	BD Biosciences	Cat #: 624296
Anti-NKp30 BB700	BD Biosciences	Cat #: 745937
Anti-Tim-3 BB660	BD Biosciences	Cat #: 624295
Anti-LAG3 BB630	BD Biosciences	Cat #: 624294
Anti-NKG7 FITC	LSBio	Cat #: LS-C377334-100
Anti-CD57 PE-Cy7	BioLegend	AB_2632689
Anti-NKG2D PE/Dazzle594	BioLegend	AB_2687173
Anti-CD158 (KIR2DL1) PE	BioLegend	AB_2130374
Anti-Siglec7 APC-Fire 750	BioLegend	AB_2814244
Anti-CD158e1 (KIR3DL1) AF700	BioLegend	AB_2130824
Anti-CD158d (KIR2DL4) APC	BioLegend	AB_2130691
Anti-DNAM-1 BV711	BioLegend	AB_2728304
Anti-CD161 BV650	BD Biosciences	Cat #: 563864
Anti-NKG2C BV480	BD Biosciences	Cat #: 748168
Anti-NKG2A BV421	BD Biosciences	Cat #: 747924
Anti-PD-1 BUV737	BD Biosciences	Cat #: 612791
Anti-CD158b (KIR2DL2) BUV661	BD Biosciences	Cat #: 750777
Anti-GM-CSF PerCp-Cy5.5	BioLegend	AB_11147946
Anti-BCL11b FITC	Abcam	AB_10973033
Anti-EOMES PE-efluor 610	eBioscience	Cat #: 61-4877-42
Anti-Tbet PE	BioLegend	AB_2200542
Anti-IFNγ AF647	BioLegend	AB_493031
Anti-CD107a BV786	BD Biosciences	Cat #: 563869
Anti-IL-2 BV650	BioLegend	AB_2563878
Anti-H3K27ac	Cell Signaling Technology	AB_10949503
Anti-H3K27me	Cell Signaling Technology	AB_2616029
Anti-H3K4me3	Cell Signaling Technology	AB_2616028
Anti-rabbit IgG	Antibodies-online	AB_10775589

**Biological samples**		

PBMC samples from study participants living with HIV-1 and HIV-1 negative individuals.	Ragon Institute Biobank and MGH Blood Bank	ragon.partners.org

**Chemicals, peptides, and recombinant proteins**		

Concanavalin-A coated magnetic beads	Bangs Labooratory	Cat #: BP531
Digitonin	Millipore	Cat #: 300410
CUTANA™ pAG-MNase for ChIC/CUT&RUN 50 Rxns	Epicypher	Cat #: 15-1016
IL-15	Miltenyi	Cat #: 130-095-764
Brefeldin A	BioLegend	Cat #: 420601
Monensin	BD Biosciences	Cat #: 554724
Venetoclax	Selleckchem	Cat #: S8048
Poly(I:C)	Invivogen	Cat #: tlrl-pic-5
LIVE/DEAD™ Fixable Blue Dead Cell Stain Kit	Thermo Fisher Scientific	Cat #: L23105
FcR Blocking Reagent, human	Miltenyi	Cat #: 130-059-901
FOXP3 / Transcription factor staining buffer set	Invitrogen/eBioscience	Cat #: 00-5523-00
Paraformaldehyde solution 4% in PBS	Affymetrix	Cat #: 4243418
Distilled, deionized or RNAse-free H2O	Promega	Cat #: P1197
Manganese Chloride (MnCl2)	Sigma Aldrich	Cat #: 203734-5g
Calcium Chloride (CaCl2)	Fisher Scientific	Cat #: BP510-100g
Potassium Chloride (KCl)	Sigma Aldrich	Cat #: P3911-25g
Hydroxyethyl piperazineethanesulfonic acid	Sigma Aldrich	Cat #: H3375-25g
Sodium chloride (NaCl)	Sigma Aldrich	Cat #: S5150-1L
Ethylenediaminetetraacetic acid (EDTA)	Sigma Aldrich	Cat #: E7889
Ethylene glycol-bis(β-aminoethyl ether)-N,N,N,N-tetraacetic acid (EGTA)	Sigma Aldrich	Cat #: E3889-10G
Roche Complete Protease Inhibitor EDTA-Free tablets	Sigma Aldrich	Cat #: 11873580001
RNase A, DNase and protease-free (10 mg/ml)	Thermo Fisher Scientific	Cat #: EN0531
Agencourt AMPure XP magnetic beads	Beckman Coulter	Cat #: A63880
Sodium dodecyl sulfate (SDS)	Sigma Aldrich	Cat #: L4509-25G
Proteinase K	Thermo Fisher Scientific	Cat #: EO0491
Phenol-chloroform-isoamyl alcohol	Thermo Fisher Scientific	Cat #: 15593031
Chloroform	Sigma Aldrich	Cat #: 366919-1L
1 M Tris-HCl pH 8.0	Fisher Scientific	Cat #: AAJ22638AP
20 mg/ml Glycogen	Sigma Aldrich	Cat #: 10901393001
Agencourt RNAClean XP	Beckman Coulter	Cat #: A63987
SUPERase⋅ In™ RNase Inhibitor (20 U/μL)	Life Technologies	Cat #: AM2696
SuperScript™ III Reverse Transcriptase	Life Technologies	Cat #: 18080085
dNTP Mix (10 mM ea)	Life Technologies	Cat #: 18427088
Ethanol 200 Proof	Decon Labs	Cat #: 2716
Poly-D-Lysine	Thermo Fisher Scientific	Cat #: A3890401

**Critical commercial assays**		

NEBNext Ultra II DNA Library prep Kit	New England Biolabs	Cat #: E7645S
NEBNext® Multiplex Oligos for Illumina® (Dual Index Primers Set 1)	New England Biolabs	Cat #: E7600S
NextSeq 500/550 High Output v2.5 kit	Illumina	Cat #: 20024906
PicoPure RNA isolation kit	Applied Biosystems	Cat #: KIT0204
Nextera XT DNA Sample Preparation Kit	Illumina	Cat #: FC-131-1096
Nextera XT Index kit	Illumina	Cat #: FC-131-1002
NK cell isolation kit	Miltenyi	Cat #: 130-092-657
Myeloid Dendritic Cells Isolation kit, Human	Miltenyi	Cat #: 130-094-487
Seahorse Glycolysis Stress Test	Agilent	Cat #: 103020-100
Quantitect SYBR Green PCR kit	Qiagen	Cat #: 204145
Qubit 1X dsDNA HS Assay Kit	Invitrogen	Cat #: Q33231
High Sensitivity D1000 Reagents	Agilent Technologies	Cat #: 5067-5585
High Sensitivity D5000 Reagents	Agilent Technologies	Cat #: 5067-5593
CyQuant LDH Cytotoxicity Assay	Invitrogen	Cat #: C20301

**Deposited data**		

Cut&Run sequencing data	This paper	Omnibus GSE232916
RNA sequencing data	This paper	Omnibus GSE232916

**Experimental models: Cell lines**		

K562	ATCC	CVCL_004

**Oligonucleotides**		

Primer: Il-15 Forward: CCGGA GATGCAAGTATTCATG	This paper	N/A
Primer: Il-15 Reverse: CCTCA CATTCTTTGCATCCAG	This paper	N/A
Primer: ACTB Forward: CTGGA ACGGTGAAGGTGACA	This paper	N/A
Primer: ACTB Reverse: CGGC CACATTGTGAACTTTG	This paper	N/A

**Software and algorithms**		

BD FACS Diva	BD Biosciences	N/A
MACS2	Langmead et al.^67^	N/A
DeepTools	Zhang et al.^68^	N/A
Diffbind	Ramírez et al.^69^	N/A
DESeq2	Risso et al.^70^	N/A
Ingenuity Pathway Analysis	Qiagen	version 90348151
FlowJo	Tree Star LLC	version 10.5.3
Wave	Agilent Technologies	version 2.6.0
QuantStudio™ Real-Time PCR Software	Thermo Fisher Scientific	version 1.3
GraphPad	Prism	version 9.5.1

**Other**		

Biorender	https://biorender.com	N/A

### Resource availability

#### Lead contact

Further information and requests for resources and reagents should be directed to and will be fulfilled by the lead contact, Xu G. Yu (xyu@mgh.harvard.edu).

#### Materials availability

This study did not generate new unique reagents.

#### Data and code availability

• The epigenomic and transcriptomic sequencing data are available in GEO and are publicly available as of the date of publication. Accession numbers are listed in the key resources table.
• This paper does not report original code.
• Any additional information required to reanalyze the data reported in this paper is available from the lead contact upon request.

### Experimental model and study participant details

HIV-1 ECs (*n* = 40) who had maintained undetectable levels of HIV-1 replication for a median of 5 years in the absence of antiretroviral therapy (viral load < 50 copies/mL; median CD4^+^ T cell count 920 cells/mm^3^), people living with HIV-1 treated with antiretroviral therapy (ARTs; *n* = 33; viral load <50 copies/mL; median CD4^+^ T cell count 727 cells/mm^3^) and people without HIV-1 (HIVNs; *n* = 31) were recruited for this study. The clinical and demographic characteristics of the study subjects are listed in Table S4. Additional PBMC samples from people without HIV-1 collected at the MGH Blood Bank were obtained for BCL-2 *in vitro* inhibition using Venetoclax experiments. All subjects gave written informed consent, and the study was approved by the IRB of Massachusetts General Hospital.

### Method details

#### Flow cytometry NK cell sorting

PBMCs were thawed, stained with LIVE/DEAD Green Viability Dye (Invitrogen) for 15 minutes at 4°C and subsequently preincubated for 10 min with of FcR blocking reagent (Miltenyi). Afterward, cells were incubated for 25 minutes at 4°C with anti-CD3 (clone HIT3a; BioLegend), anti-CD14 (clone HCD14; BioLegend), anti-CD19 (clone HIB19; BioLegend), anti-CD56 (clone HCD56; BioLegend) and anti-CD16 (clone 3G8; BioLegend) antibodies. Cells were then sorted for CD56^dim^ CD16^+^ cNK cells and CD56^bright^ CD16^-^ NK cells on a BD FACSAria Fusion (BD Bioscience) at the Ragon Institute Imaging Core Facility.

#### CUT&RUN sequencing

A standard CUT&RUN sequencing protocol was used^42^ with minor modifications. Briefly, sorted CD56^dim^ CD16^+^ and CD56^bright^ CD16^-^ NK cells from ECs, ARTs and HIVNs were bound to concanavalin A–coated magnetic beads (Bangs Laboratories), followed by cell permeabilization using 0.01% digitonin (Millipore) in wash buffer. Primary antibodies against intracellular H3K27ac, H3K27me3, and H3K4me3 histone marks (Cell Signaling Technology) were added, and cells were incubated at 4°C for overnight. Guinea pig anti-rabbit IgG antibody (Antibodies-online) was used as a negative control. The next day, fusion protein CUTANA pA/G-MNase (20×) (Epicypher) was added and cells were incubated at 4°C for 1 hour, followed by chromatin digestion using 100 mM CaCl_2_ (Thermo Fisher Scientific). Stop buffer was then added to stop the chromatin digestion. DNA fragments were extracted using phenol chloroform (Invitrogen) and the extracted DNA was eluted in 1 mM Tris-HCl pH 8.0 (Thermo Fisher Scientific) + 0.1 mM EDTA (Sigma-Aldrich). The DNA library was prepared using NEBNext Ultra II DNA Library Prep Kit for Illumina Sequencing (New England Biolabs). The DNA library concentration and quality were measured by Qubit 1× dsDNA HS kit (Life Technologies) and D1000 High Sensitivity TapeStation (Agilent), respectively. The sequencing was performed using NextSeq 500/550 High Output v2.5 kit (75 cycles) (Illumina) on a NextSeq 500 Instrument (Illumina). Adapters and low-quality reads were trimmed using Trimmomatic^71^ and aligned to the human genome (GRCh38) using Bowtie2.^67^ Peak calling was implemented using MACS2.^68^ For visualization, the coverage profile was calculated using DeepTools.^69^ Differential binding analysis was performed using DiffBind.^72^ The CUT&RUN sequencing data are available in Gene Expression Omnibus GSE232916.

#### Gene expression analysis by bulk RNA sequencing

Total RNA from sorted CD56^dim^ CD16^+^ and CD56^bright^ CD16^-^ NK cells, as well as from NK cells with and without IL-15, was extracted and purified using the PicoPure RNA Isolation Kit (Applied Biosystems). Subsequently, RNAseq libraries were generated as previously described.^73^ The whole transcriptome amplification (WTA) and tagmentation-based library preparation were performed using Nextera XT (Illumina), followed by sequencing on a NextSeq 500 Instrument (Illumina). Sequences from RNAseq were aligned to the human genome (GRCh38) using STAR^74^ and quantified using RSEM.^75^ Raw counts at gene or isoform levels were normalized using External RNA Controls Consortium spiked-in controls through RUV-seq,^70^ and then used for differential gene expression analysis with DESeq2.^76^ Transcripts per million (TPM) values were used for downstream analysis. The RNA sequencing data are available in Gene Expression Omnibus GSE232916.

#### Flow cytometry analysis

For surface marker staining, PBMCs were thawed, stained with LIVE/DEAD Blue Viability Dye (Invitrogen) for 15 minutes at 4°C, and subsequently preincubated for 10 min with of FcR blocking reagent (Miltenyi). Afterward, cells were incubated for 25 minutes at 4°C with combinations of antibodies directed against surface markers. Subsequently, the cells were fixed in 2% paraformaldehyde (Affymetrix) in phosphate-buffered saline (PBS) for flow cytometry analysis. Staining for intracellular markers and cytokines was done by resting thawed PBMCs for 2 hours at 37°C, 5% CO_2_. Cells were then co-cultured with K562 cell line or stimulated with Cell Activation Cocktail without Brefeldin A (containing PMA and ionomycin; BioLegend), in the presence of anti-CD107a antibody (clone H4A3; BD Biosciences). After 1 hour, Brefeldin A (BioLegend) and Monensin (BD Biosciences) were added, and cells were incubated for additional 4 hours. Following incubation, cells were then washed in PBS, stained with LIVE/DEAD Blue Viability Dye (Invitrogen) for 15 minutes at 4°C, and subsequently preincubated for 10 min with of FcR blocking reagent (Miltenyi). Afterward, cells were incubated for 30 minutes 4°C with combinations of antibodies directed against surface markers. Cells were then fixed and permeabilized using FOXP3/Transcription Factor Staining Buffer (Invitrogen). Next, cells were incubated for 30 minutes at 4°C with combinations of antibodies directed against intracellular markers. Flow cytometry data were acquired using BD FACSymphony (BD Biosciences) and analyzed using FlowJo v.10.5.3 software (Tree Star LLC).

#### Magnetic isolation of NK cells and mDCs

Total NK cells from PBMCs were isolated using NK Cell Isolation Kit, Human (Miltenyi) and LS columns by negative selection. mDCs from PBMCs were isolated using Human Myeloid Dendritic Cells Isolation Kit (Miltenyi) and LD columns by negative selection. The purity of cells after isolation was greater than 90%. The isolated NK cells and mDCs were used for downstream assays.

#### *In vitro* cell culture

Isolated NK cells or total PBMCs were cultured in medium only (negative control) or with 10 ng/mL IL-15 (Miltenyi) for 24 hours. When indicated, BCL2 inhibitor Venetoclax (Selleckchem) was added at a concentration of 100nM. Isolated mDCs were cultured in medium only (negative control) or with 2 μg/mL TLR3 ligand Poly(I:C) (Invivogen) for 24 hours. K562 were cultured in medium only for expansion. The cells were cultured in RPMI medium (Thermo Fisher Scientific) supplemented with 10% fetal bovine serum (FBS) (MilliporeSigma), 1% l-glutamine (Corning), 1% penicillin/streptomycin (Corning), and 1% HEPES buffer (Corning) and kept at 37°C in 5% CO_2_ during the culture.

#### *In vitro* cytotoxicity assay

Isolated NK cells were plated at 100,000 cells/well, in 50uL of medium, with or without IL-15 supplementation (10 ng/mL), as indicated, for 1h at 37°C in 5% CO_2_. After 1h, K562 cells were co-cultured with NK cells, at an effector-target range of 10:1, in a final volume of 100uL, for 4h at 37°C in 5% CO_2_. Target cell killing was analyzed by measuring LDH concentration in the culture supernatant, using the kit CyQuant LDH Cytotoxicity Assay (Invitrogen) following the manufacturer’s instructions. Cytotoxic activities were calculated using the following formula:

$$\%\;cytotoxicity=\frac{\left(LDH\;Killing-LDH\;NK\;Spontaneous-LDH\;K562\;Spontaneous\right)}{\left(LDH\;K562\;\mathrm{Max}-LDH\;K562\;Spontaneous\right)}\times 100$$

#### Glycolysis stress test

The extracellular acidification rate (ECAR), which indicates glycolytic activities was analyzed using the Seahorse Glycolysis Stress Test (Agilent) on a Seahorse XFe96 Analyzer (Agilent) according to the manufacturer’s protocol. Briefly, conditioned NK cells were harvested, washed using prewarmed supplemented RPMI medium, and plated on XF96 cell culture microplates (Agilent) coated with poly-*d*-lysine (Thermo Fisher Scientific) to adhere the cells on the microplate surface. Prior to the assay, the RPMI medium was replaced with XF DMEM Medium, pH 7.4 (Agilent), supplemented with 2 mM glutamine (Agilent). At indicated time points, ECAR was measured in the basal condition and in response to 10 mM glucose, 6 mM oligomycin and 50 mM 2-DG (Agilent).

#### RT-PCR

mDCs were washed in 1× PBS and lysed using PicoPure Extraction Buffer (Applied Biosystems). Total RNA was extracted and purified using the PicoPure RNA Isolation Kit (Applied Biosystems), according to the manufacturer’s protocol. Next, total RNA was reverse transcribed using SuperScript IV Reverse Transcriptase (Invitrogen) into cDNA. Quantitative PCR was performed using the Quantitect SYBR Green PCR Kit (Qiagen) and primers designed to amplify *IL15*. A ViiA7 instrument (Life Technologies) was used. *ACTB* was analyzed as a housekeeping gene. The expression of *IL15* relative to the negative control was calculated using the Livak (2^(–ΔΔCt)^) method.

### Quantification and statistical analysis

Differences between 2 groups were tested for statistical significance using unpaired, 2-tailed *t* test or Mann Whitney test (unpaired observations) and paired, 2-tailed *t* test or Wilcoxon matched pairs signed rank test (paired observations). Differences among 3 groups or more were tested using 1-way ANOVA or Kruskal-Wallis (unpaired observations) and repeated measures ANOVA or Friedman test (paired observations). Correlation between 2 variables was tested using Spearman or Pearson test. Normality distribution was tested using Kolmogorov Smirnov test.

## Supplemental information

Document S1. Figures S1–S4 and Table S4

Table S1. Comparison of H3K27ac, H3K4me3, and H3K27me3 mark enrichment between each cohort, related to Figure 1

Table S2. Significantly enriched H3K27ac mark and differentially expressed gene expression in EC NK cells, related to Figure 2

Table S3. Significantly enriched H3K4me3 mark and differentially expressed gene expression in EC NK cells, related to Figure 2

Document S2. Article plus supplemental information
