# Supplementary material for: IL-15-dependent immune crosstalk between natural killer cells and dendritic cells in HIV-1 elite controllers
Source: Cell Rep. 2023 Dec 3;42(12):113530. doi: 10.1016/j.celrep.2023.113530 (PMC10765318; doi:10.1016/j.celrep.2023.113530)
Supplement: Document S1. Figures S1–S4 and Table S4 [file mmc1.pdf]

Cell Reports, Volume *42*

Supplemental information

IL-15-dependent immune crosstalk between natural killer cells and dendritic cells in HIV-1 elite controllers

Ciputra Adijaya Hartana, Melanie Lancien, Ce Gao, Yelizaveta Rassadkina, Mathias Lichterfeld, and Xu G. Yu

Supplementary Figure 1

A

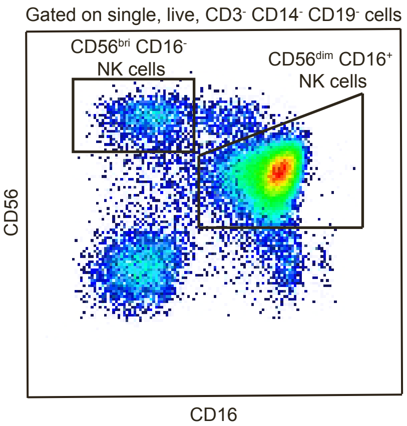

B

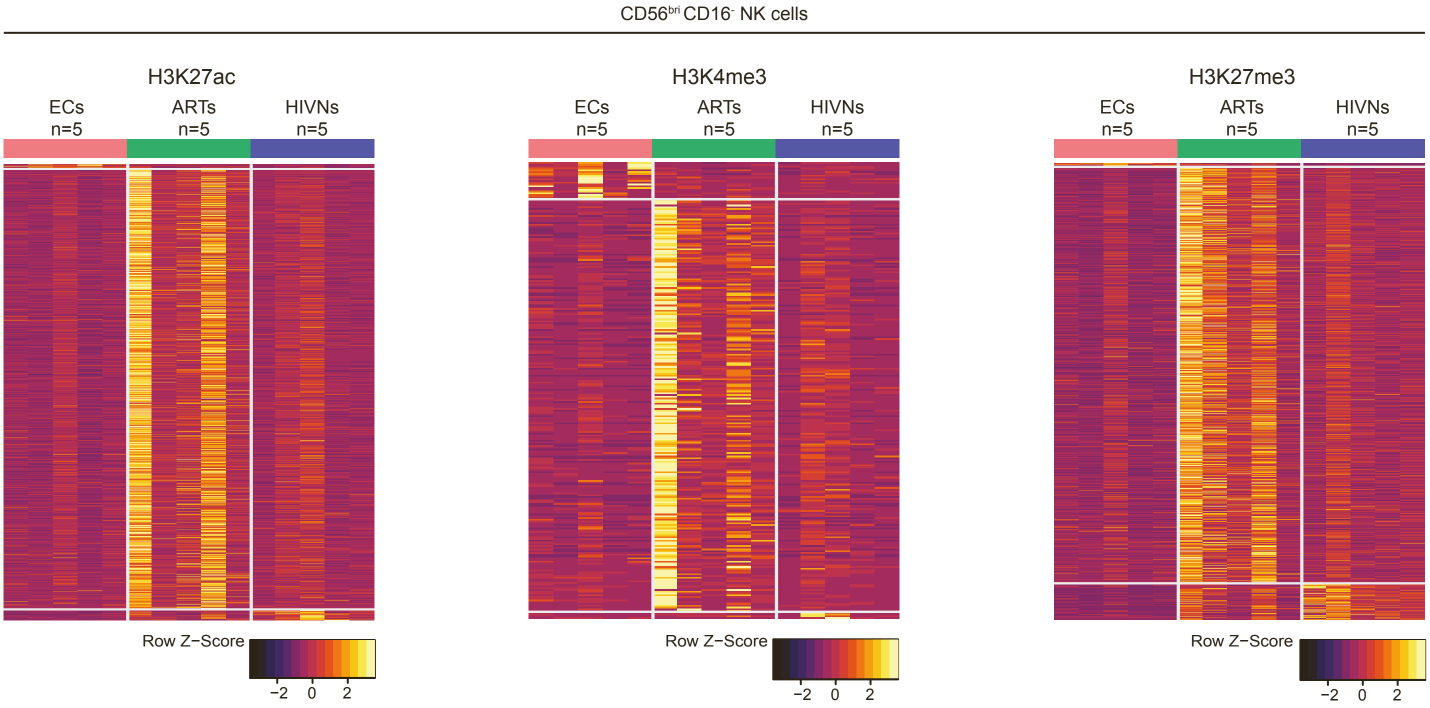

Supplementary Figure 1: Epigenomic profiles of CD56^{bright} CD16^{-} NK cells from ECs. Related to Figure 1. (**A**) Pseudocolor plot depicting the gating strategy of CD56^{dim} CD16^{+} cNK cells and CD56^{bright} CD16^{-} NK cells using flow cytometry. The NK populations were shown after gating on single, live, CD3^{-} CD14^{-} CD19^{-} cells. (**B**) Heatmap displaying genes with significantly enriched (P value < 0.05) H3K27ac (left), H3K4me3 (middle) and H3K27me3 (right) marks in ex vivo CD56^{bright} CD16^{-} NK cells from ECs (n = 5), ARTs (n = 5) and HIVNs (n = 5).

Supplementary Figure 2

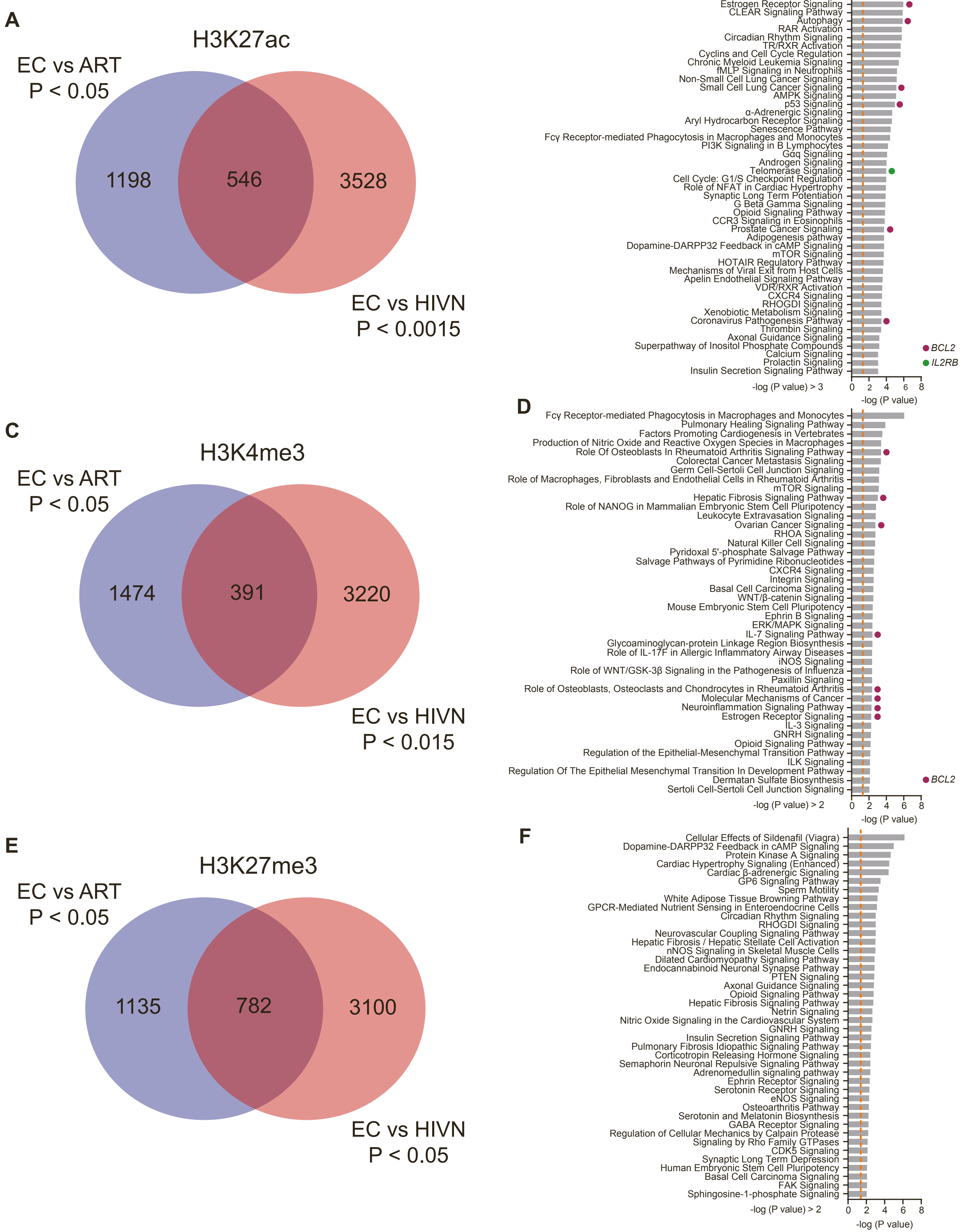

Supplementary Figure 2: Canonical pathways of genes with differential histone enrichment in CD56^{dim} CD16^{+} cNK cells from ECs. Related to Figure 1. (**A**, **C**, **E**) Venn diagrams showing the overlaps between genes with differential histone H3K27ac (A), H3K4me3 (C) and H3K27me3 (E) enrichment in primary CD56^{dim} CD16^{+} cNK cells between ECs vs. HIVNs and ECs vs. ARTs. (**B**, **D**, **F**) Significant canonical pathways predicted by Ingenuity Pathway Analysis (IPA) of overlapped genes with differentially enriched histone marks between ECs vs. HIVNs and ECs vs. ARTs from (A, C, E). Orange dashed lines marked –log (P value) = 1.3. Canonical pathways with *BCL2* and *IL2RB* were marked with purple and green dots, respectively.

Supplementary Figure 3

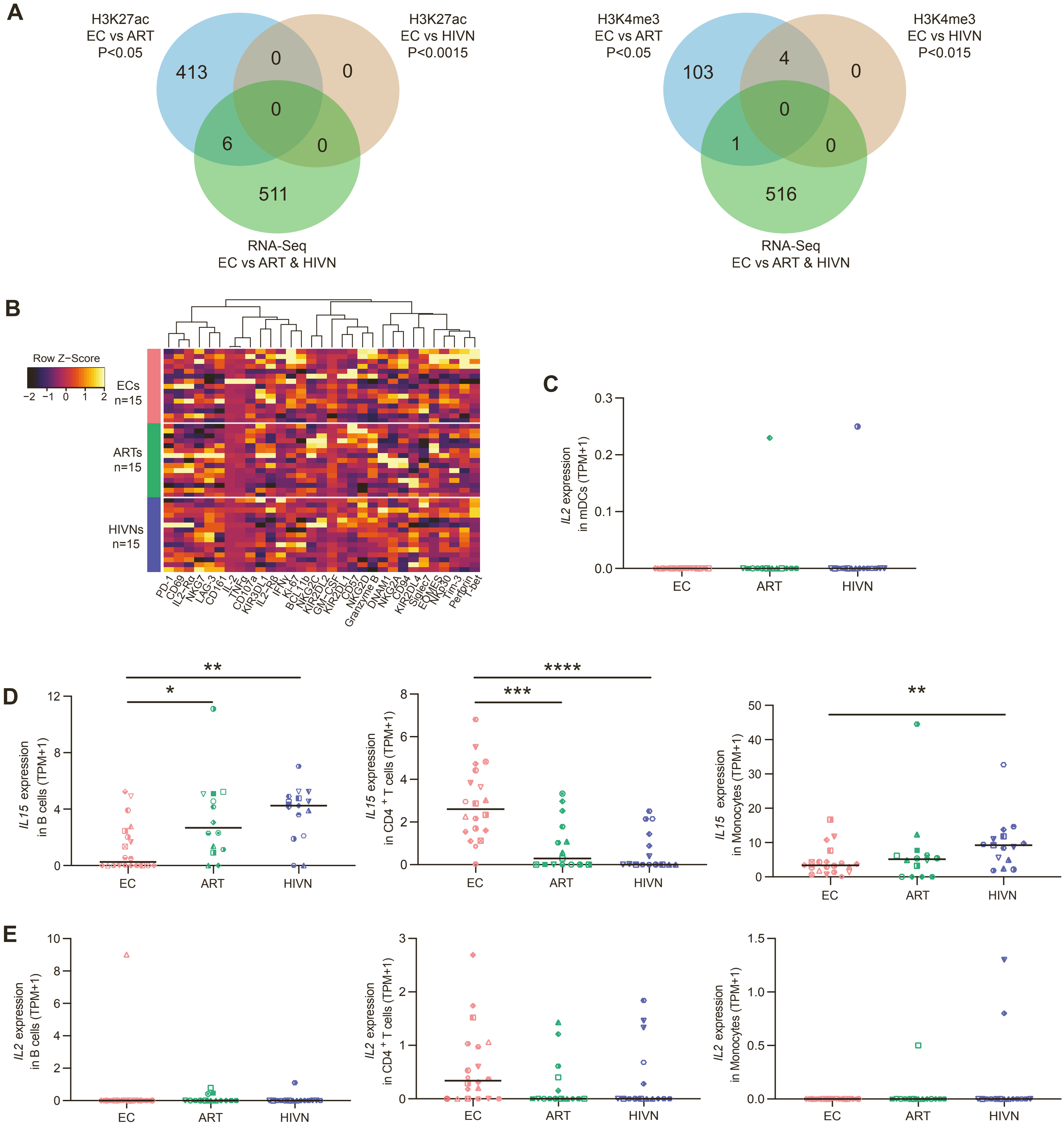

Supplementary Figure 3: mDC – NK cell crosstalk via IL-15 - IL2-Rβ axis to support trained CD56^{dim} CD16^{+} cNK cells from ECs. Related to Figure 3. (**A**) Venn diagrams showing the overlaps between genes with decreased histone H3K27ac (left) and H3K4me3 (right) enrichment in primary cNK cells between ECs vs. HIVNs and ECs vs. ARTs on the downregulated genes (P value < 0.05). (**B**) Heatmap displaying the expression of NK cell markers on the protein level in CD56^{dim} CD16^{+} cNK cells from ECs (n = 15), ARTs (n = 15) and HIVNs (n = 15), measured using flow cytometry after coculture with K562 cells. (**C**) The mRNA expression (TPM+1) of *IL2* in mDCs was compared among ECs (n = 20), ARTs (n = 13) and HIVNs (n = 15). Kruskal-Wallis test was used as the statistical test. (**D-E**) The mRNA expression (TPM+1) of *IL15* (D) and *IL2* (E) in B cells (left), CD4^{+} T cells (middle) and monocytes (right) was compared among ECs (n = 20), ARTs (n = 14) and HIVNs (n = 15). Kruskal-Wallis test was used as the statistical test. Horizontal lines in panels C-E represent medians. *P < 0.05, **P < 0.01, ***P < 0.001, ****P < 0.0001

Supplementary Figure 4

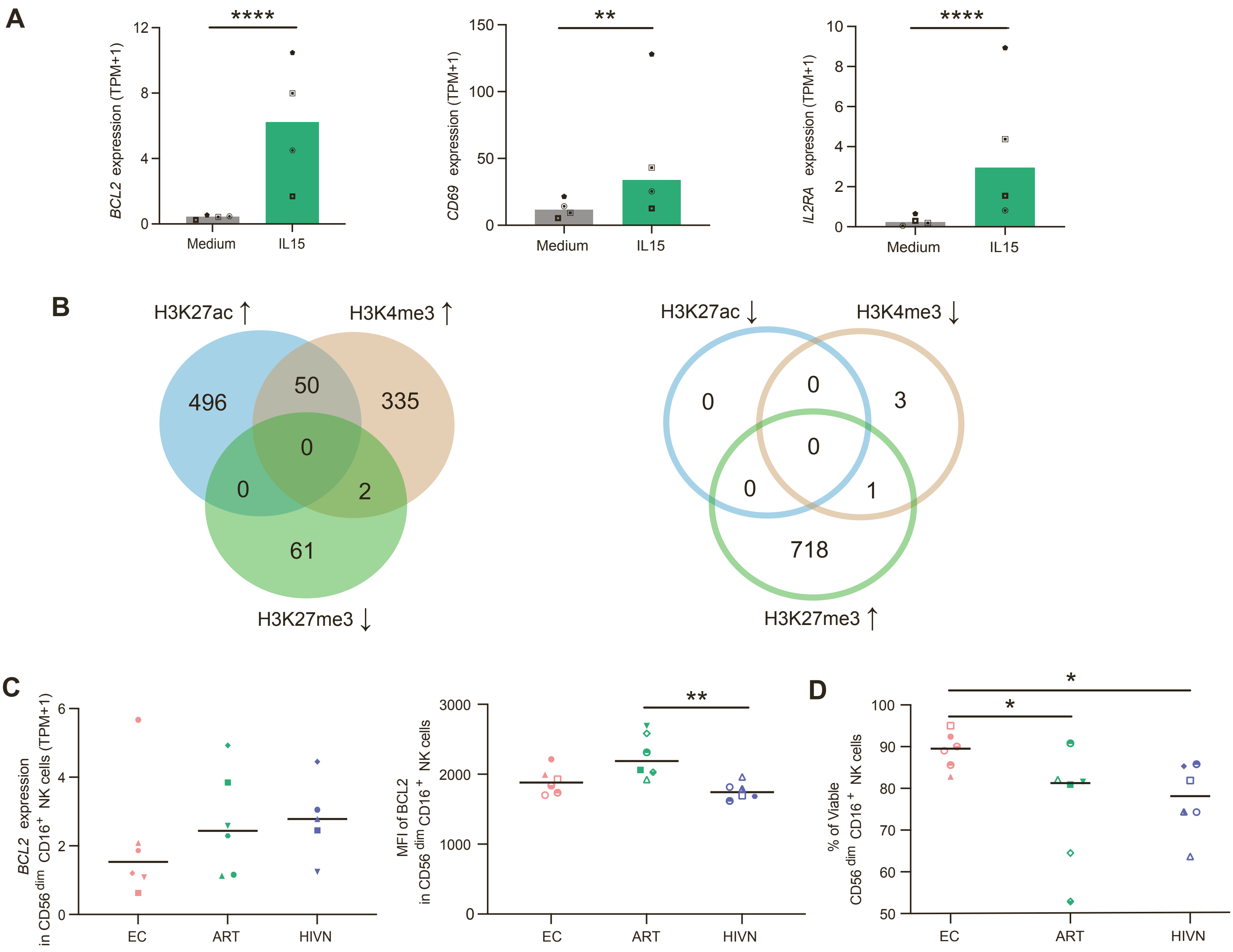

Supplementary Figure 4: Epigenetic profiles of *BCL2* gene in CD56^{dim} CD16^{+} cNK cells from ECs. Related to Figure 4. (**A**) The mRNA expression (TPM+1) of *BCL2* (left), *CD69* (middle) and *IL2RA* (right) was compared between IL-15-stimulated (n = 4) vs. unstimulated (n = 4) NK cells. Wilcoxon matched pairs signed rank test was used as the statistical test. (**B**) Venn diagrams showing the overlaps between genes with differential histone H3K27ac, H3K4me3 and H3K27me3 enrichment in primary CD56^{dim} CD16^{+} cNK cells between ECs vs. HIVNs and ECs vs. ARTs. Activation (↑H3K27ac, ↑H3K4me3 and ↓H3K27me3; left panel) and inhibitory (↓H3K27ac, ↓H3K4me3 and ↑H3K27me3; right panel) diagrams were shown. (**C**) The mRNA expression (TPM+1) (left) and the protein expression (right) of BCL2 in primary cNK cells was compared among ECs (n = 6), ARTs (n = 6) and HIVNs (n = 6). Kruskal-Wallis test was used as the statistical test. (**D**) The frequency of viable cNK cells after 10 ng/ml IL-15 stimulation were compared among ECs (n = 6), ARTs (n = 6) and HIVNs. Kruskal-Wallis test was used for statistical analysis. Bars in panel A and horizontal lines in panel C represent medians. $^{*}P < 0.05$, $^{**}P < 0.01$, $^{****}P < 0.0001$

Supplementary Table 4: Clinical and demographic characteristics of study cohorts. Related to STAR Methods.

	Elite controllers (ECs)	PLWH on ART (ARTs)	People without HIV-1 (HIVNs)
Number of participants	40	33	31
Age in years*	61 (31-75)	56 (30-72)	34 (23-68)
Female (%)‡	22.5	24.2	24.2
CD4 counts (cells/mm^3)*	905.5 (407-1684)	825 (163-1649)	N/A†
Viral loads (copies/ml)*	under limit of detection	under limit of detection	N/A†
HLA-B*27/B*57 (%)#	27.5	3	9.7

*Median with range.

‡P = 0.9485, using Chi-square test.

#P = <0.0001, using Chi-square test.

†Not Applicable
